# Editorial: Advanced biocatalysts for sustainable chemical synthesis

**DOI:** 10.3389/fmicb.2026.1862916

**Published:** 2026-05-11

**Authors:** Madan L. Verma, Deniz Yildirim, Christopher N. Boddy

**Affiliations:** 1 Department of Biotechnology, School of Basic Sciences, Indian Institute of Information Technology, Una, Himachal Pradesh, India; 2 Department of Chemical Engineering, Faculty of Ceyhan Engineering, Cukurova University, Ceyhan, Adana, Türkiye; 3 Department of Chemistry and Biomolecular Sciences, University of Ottawa, Ottawa, ON, Canada

**Keywords:** aminolevunic acid synthase, biocatalyst, D-glucuronyl C5-epimerase, enzyme engineering, keratinase, vanillyl oxidases

The need for chemical production through sustainable technologies is a major thrust area of research across many governmental organizations globally to mitigate the environmental issues and promote green-approaches (Paquette et al., 2024; Dhanya et al., 2020). Here the role of enzyme technology is crucial as it provides a platform to provide tailor-made biocatalysts for diverse biotechnological applications ranging from the environment to medicine (Tülek et al., 2025). However, the native biocatalyst is prone to external conditions such as extreme temperature and pH that render them unstable. Here, the role of enzyme engineering, particularly directed evolution, rational design, and gene editing tools, is of immense importance to tailor their biocatalytic performance in terms of specificity and activity (Paquette et al., 2024; Huguenin-Dezot et al., 2019). Further, nanotechnology-driven nanocarrier provide further stability and recyclability to make the industry process more cost-effective and desirable (Tülek et al., 2023; Verma et al., 2023). Enzyme technology supports the Sustainable Development Goal such as SDG 6, SDG 7, SDG 9, SDG 12, SDG 13, SDG 14 (https://sdgs.un.org/goals), and that is why it needs special attention to strengthen R&D of enzymes to meet diverse issues. Enzyme are vital biocatalyst that sustain life and play a significant role in biocatalysis, a mature technology that has grown with the advent of omics technology and nanotechnology approaches (Verma et al., 2019). In this Research Topic, advances in biocatalysts, including the microbial keratinases, vanillyl oxidases, aminolevunic acid synthase, D-glucuronyl C5-epimerase, and CRISPAR-CAS9 based editing *Bacillus methanolicus* bacterium to improve the function catalytic capability, have been incorporated, and are detailed in the next section.

Chu et al. highlighted the advances of microbial keratinases in tackling recalcitrant keratinaceous waste materials emanating from the leather and poultry industries. The tough waste materials, composed of excessive cross-linked disulfide bonds, pose serious environmental challenges and are difficult to process using the conventional proteases. Hence, a specific biocatalyst, keratinase, is the key player in biotransforming waste into a circular economy. However, the genuine biocatalyst was not efficient enough to achieve massive biotransformation. Hence, through the advent of genetic engineering tools, structural and functional properties were tailored to meet the targets of environmental remediation along with the industrial product. Researchers concluded that the research gap in utilizing robust keratinases for waste material processing was addressed through the use of robust keratinase biocatalysts via diverse advancements including heterologous expression, artificial intelligence (AI)-assisted design, and muti-enzyme synergistic tools, to meet industrial requirement for bioprocessing various keratinaceous waste into value added products.

Khider et al. have employed the genome editing tools, namely CRISPAR-CAS9, to improve the industrially significant thermophilic bacterium *Bacillus methanolicus* MGA3. Researchers constructed the pCasPP plasmid for efficient genome editing of target genes, namely *katA, ald*, and *spo0A* genes. Cas9 and sgRNA were induced to target double-strand breaks. Temperature regulation was selectively applied to the cleavage activity of Cas9 at 40 °C while 50 °C was utilized for DNA repair and further growth. The author reported the DNA repair mechanisms via two routes, namely homology-directed repair, and error-prone end-joining. Most interesting, three specific genes *katA, ald*, and *spo0A*, coding for catalase, alanine dehydrogenase, and sporulation regulator respectively, was validated using the genome editing tool with efficiencies exceeding 85% for deletions and 80%−95% for gene substitution. Researchers have identified limitations, such as cas9 thermal sensitivity, secondary mutations, metabolic buffering/redundancy, and fitness cost that require further investigations.

Weindorf et al. have expanded the catalytic versatility of vanillyl alcohol oxidases by discovering a subfamily that is active on para-alkylated phenol derivatives to produce ketones. Alcohol oxidases are the important oxidoreductase class that catalyses the variety of phenol derivatives to alcohol, aldehydes, and ketones. However, beginning with para-alkyl substituted phenols, the ketones are often not accessible due to oxidases not functioning toward chiral benzylic alcohols. Researchers employed phylogenetic and amino acid clustering approaches to explore one clade having a significantly different active site constitution while retaining essential residues for catalysis. Researchers have chosen five candidates from this clade for investigation. One member, namely vanillyl oxidase (VAO) from *Paecilomyces variotii* (PvVAO), was found to possess unique properties that enable it to catalyse para-alkylated phenolic compounds to ketones. A new subfamily of fungal VAOs lacks the octamerization loop compared to existing known VAOs. These new VAOs possess unique enzymatic activities that catalyse the oxidative deamination of phenolic amines at pH > 9.0 and moreover, oxidize para-alkyl substituted phenols to valuable flavor ketones compounds. However, researchers identified areas such as lower specific activities, inactive proteins production, narrow pH stability and unique behavior of lacking ether cleavage. Researchers pinpointed that the low activity with standard substrate indicates the enzyme may have evolved for bioconversion of other substrate that are yet to be identified as natural compounds.

Yang et al. have demonstrated a bioprocess for the production of a non-protein amino acid, namely 5-Aminolevulinic acid (5-ALA). Since 5-ALA is widely used in medicine, food, and agriculture industries, researchers employed a growth-coupled selection strategy to improve 5-aminolevunic acid synthase (ALAS) sourced from *Rhodobacter capsulatus* using random and site-directed mutagenesis. The derived ALAS mutants were then introduced into the GRAS organism *Corynebacteriun glutamicum*. The best mutants yielded 1.18-fold higher product compared to the wild type. Bioprocess optimization led to the maximum production of the amino acid with a concentration of 8.72 g/L in 60 h. The fermentation optimization depended on key components such as dissolved oxygen, Fe^2+^, and glycine, which rendered maximum enzyme activity. Researchers successfully demonstrated product formation with the scope for further improvement in the present study, such as a better understanding of selection pressure bias in terms of lack of correlation between 5-ALA titres in liquid culture with biomass accumulation, better downstream processing for porphyrin production responsible for hampering 5-ALA accumulation, and finally, oxidative stress to the host organism of higher product formation.

Song and Guo developed an effective methodology for the SUMO-fused recombinant protein expression system of human D-glucuronyl C5-epimerase (Glce) with a dimer structure in *E. coli* for better folding and solubility. The recombinant Glce is a key enzyme for the production of heparan sulfate proteoglycan. Researchers performed two N-terminal truncations, one at Glce167-617 and other at Glce102-617. However, the shorter construct Glce167-617 provided a higher protein expression level and purity of 98% compared to the longer constructs Glce102-617. Further, Researchers investigated the structural analysis of the protein Glce167-617 using X-ray crystallography and confirmed that the dimeric nature of protein in solution is due to the C-terminal alpha-barrel domain. Further, the enzyme retained evolutionary conserved substrate binding sites. However, researchers confirmed purity issues with the longer construct, with purity up to 50% that indicating not all the truncations are easy to fully isolate using the present methodology and require further research extensions to address such issues.

To conclude, the studies discussed in this Research Topic highlight the ongoing trends in the enzyme engineering, where the role of microbial keratinase, vanillyl oxidases, aminolevunic acid synthase, D-glucuronyl C5-epimerase, and CRISPAR-CAS9-based editing of thermophilic bacterium is excellently demonstrated. Thus, it may be concluded that the role of enzyme engineering in sustainable biochemical catalysis is rapidly expanding field that meets the sustainable development goals and addresses environmental challenges.
